# Humanization of pediatric care in the world: focus and review of existing models and measurement tools

**DOI:** 10.1186/s13052-017-0394-4

**Published:** 2017-08-30

**Authors:** Marina Tripodi, Maria Anna Siano, Claudia Mandato, Anna Giulia Elena De Anseris, Paolo Quitadamo, Salvatore Guercio Nuzio, Claudia Viggiano, Francesco Fasolino, Annalisa Bellopede, Maria Annunziata, Grazia Massa, Francesco Maria Pepe, Maria De Chiara, Paolo Siani, Pietro Vajro

**Affiliations:** 1 Cattedra di Pediatria, Dipartimento di Medicina, Chirurgia e Odontoiatria “Scuola Medica Salernitana”, Università di Salerno , Via Allende, Baronissi (Salerno), 84081 Italy; 2 Pediatria Sistematica AORN “Santobono-Pausilipon”, Naples, Italy; 3 Pediatria AOU “S.Giovanni di Dio e Ruggi D’Aragona”, Salerno, Italy

**Keywords:** Humanization, Pediatric care, Family centered care, Child-friendly healthcare

## Abstract

**Background:**

The term “humanization” indicates the process by which people try to make something more human and civilized, more in line with what is believed to be the human nature. The humanization of care is an important and not yet a well-defined issue which includes a wide range of aspects related to the approach to the patient and care modalities. In pediatrics, the humanization concept is even vaguer due to the dual involvement of both the child and his/her family and by the existence of multiple proposed models.

**Objective:**

The present study aims to analyze the main existing humanization models regarding pediatric care, and the tools for assessing its grade.

**Results:**

The main Humanization care programs have been elaborated and developed both in America (Brazil, USA) and Europe. The North American and European models specifically concern pediatric care, while the model developed in Brazil is part of a broader program aimed at all age groups. The first emphasis is on the importance of the family in child care, the second emphasis is on the child’s right to be a leader, to be heard and to be able to express its opinion on the program’s own care. Several tools have been created and used to evaluate humanization of care programs and related aspects. None, however, had been mutually compared.

**Conclusions:**

The major models of humanization care and the related assessment tools here reviewed highlight the urgent need for a more unifying approach, which may help in realizing health care programs closer to the young patient’s and his/her family needs.

**Electronic supplementary material:**

The online version of this article (10.1186/s13052-017-0394-4) contains supplementary material, which is available to authorized users.

## Background

“Humanization of care” means the process in which the patient is placed at the center of care, with the complete taking charge of this person, in respect of his feelings, of his knowledge, and of his beliefs about their health. Actually, the patient object of care becomes a subject who participates in and share the therapeutic program. In pediatrics, the Humanization of Care is intended to provide a service focused not only on the child as a patient, but necessarily also on the entire family, which is involved in the hospital reception, diagnosis and treatment phases, and physical and psychosocial processes. Knowledge in this field is constantly evolving [1, 2].

The approach to the humanization of care varies in different cultures, based on historical, ethical, religious and economics of each person. In particular, while recognizing the cross-cutting principles, the humanization interventions are often born from a specific country needs.

Lacking other similar studies, here we examined and paralleled characteristics and assessment tools of the major humanization of care existing models. This will hopefully make available useful information for realizing health care programs closer to the needs of the young hospitalized patient and his/her family.

## Models of humanization of care

The main Humanization of Care programs have been elaborated and developed in the Americas, particularly in Brazil, the USA, and Europe, with seemingly different ways from one to another but ultimately with the same aim (Table 1).

**Table 1:** Comparison of the main characteristics of the Brazilian (NHP), the North American (PFCC) and the European (CFHC) models of humanization

National humanization policy (NHP)	Patient and family centered care (PFCC)	Child-friendly health care (CFHC)
Brasil [5]	USA [16, 17, 19, 54]	Europe [33]
*Aims*		
Enable, promote and consolidate in hospitals accredited by SUS (Sistema Único de Saúde) the creation of a humanization culture that is democratic, compassionate and critical.	• Respect and dignity. • Information Sharing. • Participation. • Partnership and Collaboration. • Negotiation	• To improve the quality of health care in term of effectiveness, efficiency and equity with attention to patient safety and his satisfaction. • Services designed for the child and his family. • Interventions focus not only on managing the child’s health condition, but also on their physical or social environment • To encourage children to exercise their right to participate.
*Methods*		
• To sensitize the hospital management • Census of the hospital situation in terms of humanized services • Development and implementation of the operational plan of humanization • Evaluation of the results of the implementation of the process of humanization	• Step 1: select a care experience • Step 2: establish the “Care Experience Guiding Council” • Step 3: evaluate the current state using shadowing • Step 4: expand Guiding Council into working group and care team • Step 5: write the history of “ideal experience” • Step 6: identify projects and form project improvement teams	• Interventions in five areas: participation, promotion, protection, prevention and provision. • Training for staff. • To assist children to become “knowledgeable patients”. • To achieve synergy between: policy makers from different sectors; commissioners, providers and regulators of services; health, education and social-care organizations. • “Child-friendly” healthcare environment. • Age-appropriate interventions to reduce fear, discomfort and pain.
*Instruments*		
1. National Network for the Humanization 2. Working groups	• Family Centered Rounds (FCR) • Interdisciplinary care	• Practical model of policy based on children’s rights. • Applying evidence-based and user-friendly guidelines for health professionals and families.
*Result indicators*		
1. Welcome and user support 2. Work professionals’ work 3. Logic of management	1. Staff satisfaction 2. Parents satisfaction 3. Level of anxiety in parents and patients 4. Timing of discharge	1. Improved health 2. Reducing inequalities 3. Creating a sustainable system within the limits of available resources.

### Brazil: The National Humanization Policy (NHP)

In Brazil, large social disparities and the difference between types of hospitals (including their setting in large cities and suburbs) have determined the need to create a government task force to realize Humanization programs that were aimed at ensuring equal reception opportunities and care for all citizens. The Brazilian Federal Constitution of 1988 established a new legal basis for health policy, defining health as a right of every citizen and, therefore, an obligation of the State.

In that Country, the belief began to spread that health is a concept much wider than the mere disease’s absence, and it must include a complete physical, mental and social well-being as, indeed, had already established the WHO (World Health Organization) in 1946. Hence, given the State obligation to provide health protection, the need to establish equitable social policies was born. This led, in 2001, to the birth of the “National Program of Humanization of the Hospital” (PNHAH) [3].

The PNHAH aimed to improve the hospital care quality for all age groups, focusing primarily on the relationship between users and health professionals, among the professionals themselves, and between the hospital and the community, to ensure the best possible functioning of their Unique Health System (SUS).

Since then, the Humanization of care has been the subject of other initiatives and actions of the SUS, and what initially was a program became, in 2003, a policy: the NHP [4, 5].

All this was planned in order to create a cross-humanization culture, through the development and implementation of programs in hospitals, that included the awareness of managers and staff training, accrediting the virtuous structures as “Humanized Hospitals” .

In summary, the program aims to improve hospital reception and the patient’s care of every age, social class and their families, providing compassionate, democratic and effective cures.

The NHP is based on three principles:

- Transversality, indicating the expansion of communication between individuals and services.
- Inseparability between care and management
- Co-responsibility in the promotion and production of the health of individuals and communities.

In the Brazilian medical literature, there is currently much debate about the concepts and practices of humanization [6, 7]. In fact, the studies that brought about the opinion sand perceptions concept of humanization [8–12] overcome those which describe the humanization interventions carried out. [1, 13, 14].

### USA: Patient and family centered care

In the USA, the term humanization refers to specific interventions in the method of delivery care in different age groups. Until the first half of the twentieth century, children were admitted in the hospital without their parents for long periods [15]. Patient- and family-centered care (PFCC) emerged as a concept only during the twentieth century second half, at a time of increasing awareness of the importance of meeting the psychosocial and developmental needs of children and the families role in promoting the health and well-being of their children [16].

The concept of Family Centered Care (FCC) in paediatrics is based on the recognition that the family is the primary source of strength and support for the child and that the views of the child and family are important for making decisions about the care program [17].

The concept of PFCC has long been associated with home care: in 1992 it was founded by the “Institute for Family- Centered Care” (now "Institute for Patient- and Family-Centered Care") to encourage the development of partnerships between patients, families and healthcare providers, and to offer leadership to encourage the practice of the PFCC as well [16].

The American Academy of Pediatrics (AAP) recommends pediatric care being “accessible, continuous, comprehensive, family-centered, coordinated, compassionate and culturally effective.” Accordingly, the PFCC is defined as *“an innovative approach to the planning, delivery, and evaluation of health care that is grounded in a mutually beneficial partnership among patients, families, and providers that recognize the importance of the family in the patient’s life”* [16, 18].

The model and the principles of PFCC have been adopted and applied by other associations such as the “Children with Special Health Care Needs” (CSHCN), the “Maternal and Child Health Bureau” (MCHB), and the “Institute for Patient- and Family-Centered Care” (IPFCC), recently compared [19].

The mutually beneficial collaboration between patients, family, and provider during hospitalization is well exemplified by the Family-Centered Rounds (FCR) which consist of an *“interdisciplinary work at the bedside in which the patient and* his/her *family share control of the management plan as well as in the evaluation of the process itself”* [20].

The AAP also recommends that conducting attending rounds in patients’ rooms in the presence of family members should be a standard hospital practice, and plans on the decision of the patient’s care should be made only after such rounds, to incorporate family involvement in decision-making [21]. The FCR have the potential to create a “patient-centered” environment, to improve medical education and, in parallel, patient care, and outcome [20].

The FCR patient care and the education of students take place simultaneously. For the optimal success of the FCR and why these can benefit both patients and their families, doctors, and trainees, it is important that the hospital is equipped, also, from the space point of view [22].

There is currently no tool that is universally accepted to “measure” the implementation and results of the PFCC model [23]. However, the family-centered approach appears to significantly increase the degree of the young patients’ parents/caregivers satisfaction [24]. Despite the spread of PFCC and the AAP recommendations, the recent study of Azuine et al. noted that, based on what is reported by parents, only 2/3 of American children have received indeed a care according to this model. Notably, exclusion was predominant in underserved and uninsured families [25].

In the 2007 National Survey of Children’s Health, conducted in the USA, a considerable part of the parents reported that their child needed a better coordination of care than what they had received. Again, this was mainly reported by blacks and Latino parents and parents of children with special care needs. It follows, therefore, that the improvement and promotion of family-centered care should be implemented to help reduce the racial/ethnic disparities [26].

The pediatric Hospital for Sick Children (SickKids) in Toronto adopts the Child and Family-Centered Care (C & FCC), an approach similar to PFCC involving all processes of care. The word CARE is intended as Clinical-practice; Administration; Research; and Education, extending beyond the hospital, in the community, and in the health system. SickKids interacts locally, nationally and internationally, to give medical support and provision of services [27].

A concept in harmony and complement to the PFCC is the “Family-oriented care”, indicated by the AAP also as “Family pediatrics”, which aims to expand the pediatrician’s responsibility in having keenness to extend the medical evaluation also to the parents to identify any physical, psychological, social, that may adversely affect their children’s health [17, 28].

### Europe

#### Child friendly health care

In Europe, humanization’s policies of pediatric care were based mainly on children’s rights. Although these have been well expressed in the United Nations Convention on the Rights of the Child (UNCRC, ratified in 1989 in New York from 140 countries), many difficulties are still encountered in their implementation, and, over the years, the challenge has always been to translate these principles into a practical model. Several organizations worldwide have adopted the articles of the UNCRC in various areas of pediatric care. Among the projects promoted to implement in practice the principles of the UNCRC, the “Child-Friendly health care Initiative” (CFHI) was created in the UK in 2000 and promoted by CAI (Child-health Advocacy International), in collaboration with UNICEF (United Nations International Children’s Emergency Fund) and WHO. This initiative aims to minimize the fear, anxiety, and suffering of children and their families, through the support and the practice of 12 Standards (Additional file 1: Table S1) [29]. The main results obtained in some countries include development and integration of therapeutic play; participation of parents in the care and visit rounds; realization of multidisciplinary working committees, with the representation of parents [30]. In Bosnia and Herzegovina 13 hospitals have been awarded the title of “child-friendly” [31]. CFHI initiative introduces the concept of Child-Friendly Healthcare (CFHC), perceived as the best possible medical care for the child and not referring to any organ of formal accreditation [32].

The CFHC has recently become a real health policy as expressed in the Guidelines of the Council of Europe, elaborated by the Committee of Ministers in 2011, concerning child-friendly health care [33].The guidelines were created to offer a practical tool to the governments of the Member States for adoption, implementation, and monitoring of child-friendly health care strategy. The CFHC model was definitely "a focus on children's right health policy, on their needs, characteristics, activities and developmental capacities, and taking account of their opinions." It includes also the notion of “family-friendly” to emphasize the importance of contact between the child and his/her family as part of the care pathway.

Following to the publication of the Guidelines, the "British Association for Community Child Health" adapted the model to the economic and political framework of the UK calling it “The Family-Friendly Framework”, for the design, development, and delivery of services for children and families [34].

The principles behind the CFHC is based on participation of the child in all levels of decision-making, according to the age and degree of maturity. The prevention to avoid future health, social or emotional problems; promotion of health and its determinants; protection of children from harm are included as well, along with the efficient performance of services contributing to health and well-being of children and families.

A large survey conducted by the Committee of Ministers of the Council of Europe has shown, with 2257 children from different European countries, that there is a greater need to listen and respect in their contacts with health professionals [35]. It was born, therefore, the necessity of a health system taking into account, the needs, feelings, and opinions of pediatric patients.

Some studies analyzing the causes of the child approach inconsistent with the guidelines have found scarce health worker training in communication with the children, a factor negatively affecting their participation [36]. Others stressed that the participation of children in the medical decision-making process places them in the role of holders of rights and duties as well as responsibility bearers. To enhance their participation in the information received by caregivers and doctors, there is the need to be as objective and consistent as possible at their level of mental and relational development, in order to positively influence the decision-making process [37]. The realization of CFHC model requires significant investments in the social determinants (about 85% of total costs) and health determinants (about 15% of total costs) as well. In times of austerity, it is essential to outline the contribution to the economy of health care realization suitable for children. The application of the classical models of the economy is technically difficult because child care is often complex and less standardized [38].

#### TAT- the think and action tank on Children’s right to health

The Think and Action Tank (TAT) on Children’s rights to health is an international working group, set up in June 2013. It is a global, open network of professionals, policy makers, people working for children and supported by EPA (European Paediatric Association), which has produced a document (a rights- and equity-based platform and action cycle to advance child health and well-being) in which it is proposed a general model of implementation of the child’s right to health, which has not yet been implemented.

This document aims to introduce an operational model to prepare the institutions, organizations, policy-makers, professionals and those working for children to translate into practice the principles of child rights. In order to develop an organic model, the proposed platform must be anchored to a solid foundation, based on the rights and equity, represented by a number of elements equally important: Child Rights, Health, States, Children’s Participation, Equity, Social Justice, and Responsibility [39].

## Tools for the assessment of care humanization

In different countries, several tools have been created and used for assessing the degree of humanization and related aspects.

- In the **USA**, in 1995, the Agency for Healthcare Research and Quality (AHRQ) has launched for the first time the program "Consumer Assessment of Healthcare Providers and Systems (CAHPS)" to cope with the lack of feedback from patients about the quality of provided health services. Over time, the program has expanded beyond its original focus on health plans to address a range of health care services and to meet the various needs of health care consumers, purchasers, health plans, providers, and policymakers.

The objectives of the program CAHPS are mainly two:

- To develop standardized surveys that organizations can utilize to collect comparable information on patients’ experience with care.
- To generate tools and resources to support the dissemination and use of comparative survey results to inform the public and improve health care quality.

The three most used CAHPS surveys are:

- “CAHPS Health Plan Survey”, interviewing those enrolled in certain health programs, [Medicaid, Children’s Health Insurance Programs (CHIP) and Medicare] regarding their experiences with the health services and ambulatory care;
- “The CHAPS Clinician & Group Survey (CG-CAHPS)”, asking patients to report their experiences of primary and specialized care received in outpatient settings;
- “The CAHPS Hospital Survey (HCAHPS)”, interviewing patients about the care received during an inpatient stay at a hospital facility.

Of the many CAHPS surveys, there are the adult version (over 18) and those for children (in which parents report the experience of a child aged 17 years old and under).

The CAHPS surveys are available in English and Spanish. The AHRQ also provides support and technical assistance to users through CAHPS User Network and CAHPS database that receives data sent voluntarily by users, and aggregates them to facilitate comparisons of the results [40].

In the USA, again, the American Medical Association (AMA) in collaboration with several other organizations developed the “Communication Climate Assessment Toolkit (C-CAT)”, a number of investigative tools that are distributed to staff, managers and patients to provide a comprehensive assessment of the organization’s communication capabilities of health care to the patient (patient- centered communication) [41].

b. In **Europe**, the picture is even more fragmented. The Task Force HPH-CA (Health Promoting Hospitals and Health Services for Children and Adolescents), established in April 2004 within the International Network of Health Promoting Hospitals, produced the SEMT (Self-Evaluation Model and Tool in respect of children’s rights in the hospital).

The specific objective of the model is to assess the gap between:

- Full respect for the rights of the child in hospital,
- Current situation

As a basis to promote the improvement and internal change through the development of standards, the adoption of measures, subsequent evaluations, and feedback monitoring gaps and producing change. The stages of this process of assessment, improvement, and change are represented by:

1. Mapping of real existing goods using a self-evaluation tool;
2. Planning for improvement through the identification of a set of standards for the respect of children’s rights in the hospital;
3. Production of improvements by implementing specific actions;
4. Evaluation of the changes by monitoring progress and gaps.

The SEMT was made available in 10 different languages and the pilot project was conducted in 17 hospitals in Europe and also in Australia. The area of the rights found to be more difficult to deal with by the hospitals is regarding the "child's right to information and participation in all decisions about his or her health care". Hospitals that have obtained the best results in terms of respect for children’s right in Europe are Tallinn Children’s Hospital (Estonia), Caldas da Rainha Hospital (Portugal), Meyer University Children’s Hospital (Italy) [42].

In 2012, the Task Force prepared a manual and new tools in order to further implement the self-assessment and improvement of the respect of the rights of children in hospitals at different levels (workers of services; health care professionals; children aged 6–11 and children/adolescents aged 12–18 years, parents and carers) [43].

### National experiences: Italy and France

The available data for **Italy** show that the AGENAS (Italian National Agency for Health Services) has recently produced a questionnaire for the assessment of the degree of humanization of care in Italian hospitals related to physical accessibility, livability and comfort of hospitals; welfare and organizational processes oriented to respect and to person’s specificity; care of the relationship with the patient and with the citizen; access to information, simplification and transparency. The checklist assesses the humanization level, addressed to a focus group composed by members of the hospital’s administration, doctors, nurses and voluntary associations together with citizen representatives. The study conducted in 2012 in 256 shelters spread all over the country shows that hospitals with > 800 beds obtained the best average results [44]. The most serious problems which emerged deals with respect for confidentiality, linguistic and religious specificities and foreign citizens’ reception, architectural or sensory barriers, booking arrangements, online access to clinical records, training of communication personnel, birth-analgesia. In general, the pediatric wards of hospitals received the best scores, but the analysis was not extended to all pediatric hospitals and only some relevant aspects of the humanization of pediatric care (such as procedural pain)were partly taken into account.

Still in Italy, for the subjective evaluation of the degree of perceived humanization in hospitals, the Politecnico of Milan has developed and tested, in 2014, the LpCp-tool (listening to people-to-cure people). The questionnaire, consisting of a small number of questions, still represents a suitable tool that addresses topics such as the comfort of the environments, the presence of green areas, patient involvement in the therapeutic process and security in the hospital. The most critical issues emerged in the wellness area (comfort of the environment, recreation and sports), safety, patient involvement in the therapeutic process and the physician in the design process (involvement in case of changes within the hospital environment). The results of the questionnaires administered to the staff, patients, and visitors to a general hospital in Milan with 600 beds showed divergent perceptions among the groups interviewed with a positive perception of patients about the efficiency of care received compared to the more realistic and critical view of the health operators [45]. These divergent perceptions were recently confirmed also in a pediatric setting pilot study in Campania Region using the same tool [46].

In **France**, since 2011, the French Ministry of Health has developed a questionnaire to assess the degree of satisfaction of patients hospitalized in health facilities that perform medical activities, surgery or midwifery. This indicator (e-SATIS) reflects the actions put in place to take care of patients: human, technical and its logistics management. Initially, questions were answered by telephone, later on (since 2015), online questionnaires have been submitted by e-mail to the patient 2 weeks after hospitalization. In 2014, 877 facilities were involved and 5900 patients contributed to the national results of evaluating the following aspects: global patient care, doctor’s attitude, patient and healthcare communication, information and comfort of the rooms. The last two areas were the most deficient. The aim is to help improve the quality of health services as near as possible to patients’ expectations [47].

## Discussion and recommendations

The humanization of pediatric care presupposes interventions in different areas, e.g. child-friendly environment, patient medical relationship, technology, etc. The patient-centered approach is one of the ways of understanding humanization of care, according to the American model. Although at present there is no structured meta-analysis of RCTs evaluating and comparing the outcome of humanization interventions aiming to improve pediatric care, the literature overall [48] seems to support the view that adopted interventions may have beneficial effects on several outcomes of the cure, e.g. Family centered rounds and discharge timing [49] and family satisfaction [50], programs for staff training [51]. Limited data in several fields diminish the strength of recommendations, and in many cases clinical judgment alone therefore continues to be paramount.

Nowadays, the humanization of care, is considered an aspect that cannot be overlooked, but it still receives not all the attention it deserves, with scarcity of data on the level of humanization of pediatric structures that have been properly evaluated, and “humanization patterns” often not translated into practice.

The reasons for this can be many and different depending on the circumstances of each health setting. One aspect that is likely to “hinder” the adoption of this approach is the small space given to the topic of humanization during the university education of physicians and healthcare professionals (there is no specific course of “humanization of care”). It is necessary to move to a holistic view of the patient from the evaluation of the disease itself to the evaluation of the disease in the context of the person and of the daily life. In pediatrics, this implicates the necessary involvement of the family as an active part of the care program.

Attention to the humanization aspect can probably improve the quality of care offered and consequently the satisfaction of the users of the assistance received. Especially in our country, the attention and improvement of the degree of humanization of care can be a useful tool to limit the vast South-to-North extra regional migration. Pediatric migration is, in fact, an important phenomenon with obvious and multiple implications: in addition to causing stress for patients and their families, it results significant costs for the native Region by subtracting, at the same time, economic resources for the development of human resources and for the technological upgrade [52].

Potential levels to use to implement humanization measures could be the following: [39]

A) Basal evaluation of the grade of humanization of the hospital / outpatient setting
B) From the previous assessment, identify the deficient aspects in terms of humanization on which to act
C) Raise awareness and training in hospital management and nursing staff
D) Undertake improvement interventions
E) Evaluate post-intervention efficacy

It is always advisable to look at the patient in its totality, regarded as a person and not just to the illness he is suffering from. In pediatrics this implies the evaluation of the child and his illness in the context in which he lives, considering the family as an integral part. Parents or caregivers should be considered important partners of the child care clinic, making them part of the care program and the decisions to take. Since hospitalization is a trauma, especially in childhood, the hospital should be made as much “child - friendly” as possible, with adequate furniture, spaces that recall the home environment and facilities for the parents’ child care h24.

## Conclusions

**Fig. 1:**
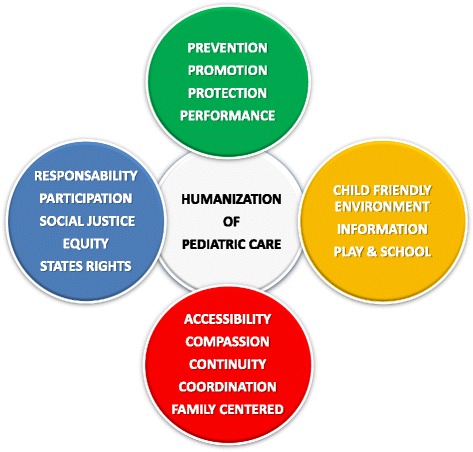
Synopsis of the principles of humanization of pediatric care expressed by/in different associations and international documents (AAP, TAT, UNCRC, CFHC) Colors and abbreviations used: Red: American Academy of Pediatrics (AAP); Green: Child Friendly Healthcare [(The United Nations Convention on the Rights of the Child (UNCRC)]. Light blue: Think and Action Tank (TAT) on the Child Right to Health (EPA European Pediatric Association). Orange: 12 Standards of the Child Friendly [(UNO-UNCRC)(CFHC child-friendly healthcare)]

The term “humanization”, in pediatrics, includes a wide range of meanings and aspects which are related to the care of the child hospitalized and not. In general, it refers to policies/measures intended to ensure accessibility and equality of treatment for all children, regardless of social class, nationality, religion, etc. Our study showed that the examined models, though acting in different ways, do share some common principles, including the involvement of the child and the family and the recognition of the children’s rights to an environment that suits their needs, limiting the trauma of the disease as possible and the suffering (Fig. 1).

Pending a universally agreed humanization definition and the spreading of policies, efforts for humanization of structures and activities are necessary to improve the period of the child’s hospitalization and his/her family through locally implemented actions. The efficacy of such variegated local actions often differ from country to country [53]. However, this does require proper evaluation to standardize and optimize as much as possible the quality of pediatric care measures. Agreement on a limited number of well-validated assessment tools appears urgently needed.

## Additional file

Additional file 1: Table S1. (online only). 12 Standards of Child Friendly Healthcare Initiative (CFHI). (DOCX 23 kb)

## Abbreviations

AAP: American Academy of Pediatrics
AGENAS: Agenzia Nazionale Italiana per I Servizi Sanitari
AHRQ: Agency for Healthcare Research and Quality
AMA: American Medical Association
C & FCC: Child and Family-Centered Care
CAHPS: Consumer Assessment of Healthcare Providers and Systems
C-CAT: Communication Climate Assessment Toolkit
CFHC: Child-Friendly Health Care
CFHI: Child-Friendly Healthcare Initiative
CHIP: Children’s Health Insurance Programs
FCC: Family Centered Care
FCR: Family Centered Rounds
LpCp-tool: Listening to people to cure people
NHP: National Humanization Policy
PFCC: Patient and Family Centered Care
PNHAH: Programa Nacional De Humanização da Assistência Hospitalar
UNCRC: The United Nations Convention on the Rights of the Child
UNICEF: United Nations International Children’s Emergency Fund
WHO: World Health Organization
