# Supplementary material for: Humanization of pediatric care in the world: focus and review of existing models and measurement tools
Source: Ital J Pediatr. 2017 Aug 30;43:76. doi: 10.1186/s13052-017-0394-4 (PMC5577665; doi:10.1186/s13052-017-0394-4)
Supplement: Additional file 1: Table S1. — (online only). 12 Standards of Child Friendly Healthcare Initiative (CFHI). (DOCX 23 kb) [file 13052_2017_394_MOESM1_ESM.docx]

| **Additional file 1: Table S1 (online only). 12 Standards of Child Friendly Healthcare Initiative (CFHI)** [29] | |
| --- | --- |
| Standard 1 | Keeping children out of hospital (and other health facilities or institutions) unless this is best for the child: |
| Standard 2 | Supporting and giving the best possible healthcare |
| Standard 3 | Giving healthcare safely in a secure, clean, “child friendly‟ environment |
| Standard 4 | Giving “child centered‟ healthcare |
| Standard 5 | Sharing information and keeping parents and children consistently and fully informed and involved in all decisions |
| Standard 6 | Providing equity of care and treating the child as an individual with rights |
| Standard 7 | Recognizing and relieving pain and discomfort |
| Standard 8 | Giving appropriate resuscitation, emergency and continuing care for very ill children |
| Standard 9 | Enabling play and learning |
| Standard 10 | Recognizing, protecting and supporting vulnerable or abused children |
| Standard 11 | Monitoring and promoting health |
| Standard 12 | Supporting “best possible” nutrition |
